# Peculiar *Paramecium* Hosts Fail to Establish a Stable Intracellular Relationship With *Legionella pneumophila*

**DOI:** 10.3389/fmicb.2020.596731

**Published:** 2020-10-23

**Authors:** Kenta Watanabe, Yusei Higuchi, Mizuki Shimmura, Masato Tachibana, Masahiro Fujishima, Takashi Shimizu, Masahisa Watarai

**Affiliations:** ^1^Joint Faculty of Veterinary Medicine, Laboratory of Veterinary Public Health, Yamaguchi University, Yamaguchi, Japan; ^2^Joint Faculty of Veterinary Medicine, Laboratory of National BioResource Project Paramecium, Yamaguchi University, Yamaguchi, Japan; ^3^Department of Research Infrastructure, National BioResource Project of Japan Agency for Medical Research and Development, Tokyo, Japan

**Keywords:** *Legionella pneumophila*, *Paramecium*, intracellular relationships, digestion, *Legionella*-containing vacuoles

## Abstract

*Legionella pneumophila*, an intracellular human pathogen, establishes intracellular relationships with several protist hosts, including *Paramecium caudatum*. *L. pneumophila* can escape the normal digestion process and establish intracellular relationships in *Paramecium*. In this study, we identify new *Paramecium* strains that significantly reduce the number of *L. pneumophila* during infection. As a result, stable intracellular relationships between *L. pneumophila* and these *Paramecium* strains were not observed. These digestion-type *Paramecium* also showed high efficiency for *Escherichia coli* elimination compared to other strains of *Paramecium*. These results suggest that the digestion-type strains identified have high non-specific digestion activity. Although we evaluated the maturation process of *Legionella*-containing vacuoles (LCVs) in the *Paramecium* strains using LysoTracker, there were no discriminative changes in these LCVs compared to other *Paramecium* strains. Detailed understanding of the mechanisms of high digestion efficiency in these strains could be applied to water purification technologies and *L. pneumophila* elimination from environmental water.

## Introduction

*Legionella pneumophila* is the causative agent of Legionnaires’ disease, which causes serious pneumonia in humans. Cases of human infection occur by inhalation of bacterial-contaminated aerosols, which are produced in artificial aquatic environments (Fliermans et al., 1981; Tachibana et al., 2013). *L. pneumophila* exists in natural aquatic environments and often establishes relationships with various species of protists (Boamah et al., 2017; Best and Abu Kwaik, 2018). Free-living amebae and ciliates, such as *Tetrahymena*, are well-known natural hosts of *L. pneumophila* (Rowbotham, 1980; Fields et al., 1984). It has also been reported that *L. pneumophila*’s relationships with protist hosts contribute to its survival in certain environments (Winiecka-Krusnell and Linder, 1999). We have previously reported that the freshwater ciliates *Paramecium* spp. could be natural reservoirs of *L. pneumophila* (Watanabe et al., 2016), and have described some mechanisms of establishment of the intracellular relationship between *L. pneumophila* and *Paramecium* (Nishida et al., 2018; Watanabe et al., 2018). However this relationship remains largely unknown.

Generally, *Parameciu*m shows high phagocytic uptake activity, and internalized bacteria are usually digested immediately after feeding. However, *L. pneumophila* is able to avoid digestion and establish intracellular relationships inside *Paramecium* food vacuoles. Previously, we have identified bacterial genes involved in the accomplishment of intracellular survival and analyzed the mechanisms of action of the respective products (Watanabe et al., 2016, 2018; Nishida et al., 2018). We have shown that these genes contribute to resistance to digestive enzyme (Watanabe et al., 2018), or to avoiding egestion by *Paramecium* hosts (Nishida et al., 2018). These previous studies suggest that *L. pneumophila* utilizes these genes and obtains the appropriate niches to survive by isolating themselves from an environment with many stress factors. In addition, the high mobility and cell division rates of *Paramecium* could be huge advantages for the spread of *L. pneumophila* in natural environments. However, it is not clear what, if any, benefits *Parameciu*m gains by harboring *L. pneumophila*. Although there is no evidence of mutualistic symbiosis between *Paramecium* and *L. pneumophila*, it is not uncommon to find that endosymbiotic bacteria provide some benefit to their protist hosts. For example, some strains of *Paramecium* develop salinity tolerance and heat shock tolerance when maintaining *Holospora* spp. within their nuclei as an obligate symbiont (Smurov and Fokin, 1998; Hori and Fujishima, 2003; Fujishima et al., 2005; Hori et al., 2008). Meanwhile, *Holospora* spp. are not essential symbionts for the host’s survival. Furthermore, maintaining these symbionts causes negative effects such as inhibition of growth, lethal cell burst, and functional failure of a micronucleus in some situations (Görtz and Fujishima, 1983; Fujishima and Kodama, 2012). It has also been reported that the endosymbionts *Neochlamydia* and *Protochlamydia* provide protection against *L. pneumophila* infection to their amebal hosts (Ishida et al., 2014; Maita et al., 2018; König et al., 2019). These findings indicate that there is a complicated relationship between hosts and parasites or symbionts.

While the stable intracellular relationship between *L. pneumophila* and *Paramecium* for at least 48 h was clearly observed in our infection assay, another type of relationship was also observed (Watanabe et al., 2016). Depending on the combination of strains of *L. pneumophila* and *Paramecium*, a cytotoxic relationship was observed; in some combinations, intracellular *L. pneumophila* killed the host *Paramecium*, thereby stable intracellular relationships were not observed (Watanabe et al., 2016). We analyzed the cytotoxic strains of *L. pneumophila* and identified the gene, *L. pneumophila* endosymbiosis-modulating factor A (*lef*A), involved in cytotoxicity toward *Paramecium* (Watanabe et al., 2016). Using *lef*A, *L. pneumophila* is able to disrupt unsuitable *Paramecium* hosts and escape from the intracellular lifestyle and revert to the free-living lifestyle. It seems it is important for *L. pneumophila* to achieve the opportunity to encounter suitable hosts. These our previous studies suggest that the relationship between *L. pneumophila* and *Paramecium* is complicated and diverse.

In this study, we hypothesized that in some cases the intracellular relationship could not be established because of digestion of *Legionella* by *Paramecium*, and attempted to identify the *Paramecium* strains showing such features (Supplementary Figure S1). We searched combinations of *L. pneumophila* and *Paramecium* strains which show the high digestion efficiency by infection tests using 60 strains of *Paramecium*. Furthermore, we analyzed the mechanisms of the *Paramecium* digestion of *L. pneumophila* by comparison to other *Paramecium* strains.

## Materials and Methods

### Bacterial Strains and Culture Conditions

*Legionella pneumophila* Philadelphia-1 (Phi-1) and Ofk308 were maintained as frozen glycerol stocks and cultured at 37°C on either *N*-(2-acetamido)-2-aminoethanesulfonic acid-buffered charcoal yeast extract (BCYE) agar or in the same medium without agar and charcoal (AYE). *Escherichia coli* DH5α were cultured in either lysogeny broth (LB) or on 1.5% LB agar. If necessary, chloramphenicol (10 μg/mL) was included in the media. Green florescent protein (GFP) expression in *L. pneumophila* was induced by adding isopropyl-β-D-thiogalactopyranoside (1 mM) to AYE.

### *Paramecium* Strains and Culture Conditions

All *Paramecium* strains used in this study were provided by the Symbiosis Laboratory, Yamaguchi University, with support from the National Bioresource Project (NBRP)^1^. *Paramecium* strains were cultured and maintained as previously described (Watanabe et al., 2016). In brief, the culture medium used for *Paramecium* was 2.5% (w/v) fresh lettuce juice in Dryl’s solution (Dryl, 1959), inoculated with a non-pathogenic strain of *Klebsiella pneumoniae*. The cultivation of *Paramecium* strains was performed at 25°C. Cells at the stationary phase of growth (24 h after the last feeding) were used for all experiments.

### Identification of *L. pneumophila-*Digesting *Paramecium* Strains

As a first screening assay, 60 *Paramecium* strains were infected with *L. pneumophila* Ofk308 in 1.5 mL tubes at an multiplicities of infection (MOI) of 10,000 according to a previously reported method (Watanabe et al., 2016) with slight modifications. Tubes were incubated at 25°C for 24 h. After incubation, samples were treated for 30 min at 50°C to purge the *K. pneumoniae* fed to the *Paramecium*. Ten microliters of each sample was spotted onto GVPC agar (BCYE agar supplemented with glycine; Wako, 3 mg/mL), containing vancomycin HCl (Wako, 1 μg/mL), polymyxin B (Wako, 80 IU/mL), and sulfate cycloheximide (Wako, 80 μg/mL). As a second screening assay, the *Paramecium* strains selected during the first screening assay were infected with *L. pneumophila* Ofk308 in the same manner as a first screening assay. Incubated samples were cultured on GVPC agar after serial dilution to determine the colony forming units (CFU). The reduction of bacterial loads were evaluated by classification into three classes: -; no reduction of bacterial load (50–100%), ±; low reduction of bacterial load (less than 50%), +; high reduction of bacterial load (less than 10%) compared to control (cultured *Legionella* in the medium without *Paramecium*).

### Determination of the Size, Food Vacuole Formation, and Cell Division Rate of *Paramecia*

The sizes of *Paramecium multimicronucleatum* Y-2, YM-25, and *Paramecium caudatum* RB-1 were observed using a FluoView FV100 confocal laser scanning microscope (Olympus). To evaluate the capacity of food vacuole formation, 1,000 *Paramecium* cells were cultured with 4.55 × 10^7^ particles of microspheres (Fluoresbrite^®^ YG Carboxylate Microspheres 1.00 μm, Polysciences) for 30 min. Cells were fixed with 4% paraformaldehyde in PBS for 10 min at room temperature. Subsequently, samples were washed twice with PBS and applied to a glass slide which had 0.07 mm gap with a cover glass (Matsunami Glass). Food vacuoles in 20 *Paramecium* cells were counted under light microscopy (Olympus, BX51). Data were represented as averages based on triplicate samples from three identical experiments. To evaluate the cell division rate, the numbers of cells at the start of culture were aligned in 100 cells/mL and cultured without adding any bacteria. After incubation at 25°C for 24 h, to count the numbers of cells again, cells were fixed with 4% paraformaldehyde in PBS for 10 min at room temperature. Subsequently, samples were washed twice with PBS and applied to a glass slide described above. With the number of cells at the start of culture defined as 100%, the cell numbers after 24 h are shown as relative values. Data were represented as averages based on triplicate samples from three identical experiments.

### Evaluation of Digestion Ability of Intracellular Bacteria

The *Paramecium* strains Y-2, YM-25, and RB-1 were infected with *L. pneumophila* or *E. coli* at MOIs of 1,000, 10,000, and 100,000 in the same manner as in the screening assay described above. After incubation at 25°C for 24 h, CFU were determined by serial dilution on BCYE or LB agar containing chloramphenicol. Data were represented as averages based on triplicate samples from three identical experiments.

### Determination of Mobility of Paramecia

The mobility (swimming velocity) of Y-2, YM-25, and RB-1 was measured according to a previously reported method (Fujishima et al., 2005) with slight modifications. Briefly, each live *Paramecium* strain was applied to a glass slide which had 0.07 mm gap with a cover glass (Matsunami Glass). This slide was placed on the stage of microscope and pictures of the cells under dark-field illumination were taken with an exposure time of 0.5 s. The lengths of the swimming loci of the cells were measured to calculate mean swimming velocities. The loci of 20 cells were measured in each. Data were represented as averages based on three identical experiments.

### LysoTracker Analysis

Green florescent protein-expressing *L. pneumophila* were added to Y-2, YM-25, and RB-1 at an MOI of 10,000 and then incubated at 25°C for 30 min or 120 h. Samples were fixed with 4% paraformaldehyde in PBS for 10 min at room temperature. Subsequently, samples were washed twice with PBS. Fluorescent images were obtained using a FluoView FV100 confocal laser scanning microscope. LysoTracker (Life Technologies) was used after fixation for 30 min at a concentration of 50 nM. Data were represented as averages based on triplicate samples from three identical experiments.

### Statistical Analysis

Statistical analyzes were performed using Student’s *t*-test in Figures 2, 4, 5 or the Tukey–Kramer test in Figure 3. Data comprised the averages of three identical experiments. Standard deviations were represented as error bars.

## Results

### The Identification of *Paramecium* Strains Showing High Digestion Efficiency

**FIGURE 1:**
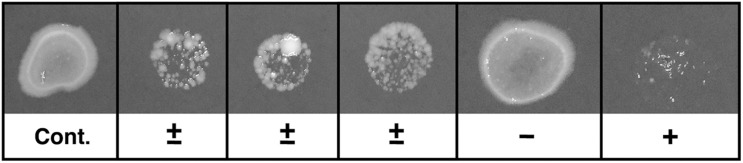
Typical example of screening test. Ten microliters of each sample were spotted onto GVPC agar. The agar plates were incubated at 37°C. The relative colony volumes were evaluated by comparison with the control (without *Paramecium*). Control and typical example of samples were evaluated as -; no reduction of bacterial load (50–100%), ±; low reduction of bacterial load (less than 50%), +; high reduction of bacterial load (less than 10%) compared to control.

**FIGURE 2:**
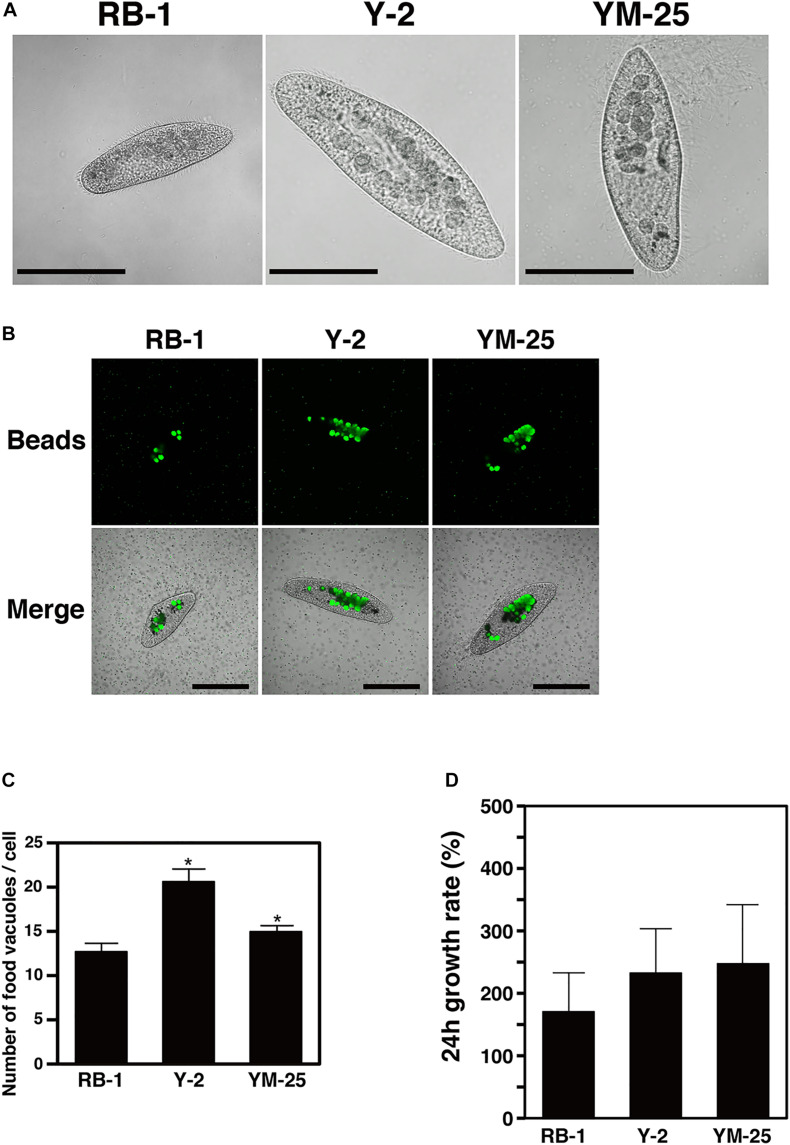
The size, food vacuole formation, and cell division rate of Y-2 and YM-25 strains. **(A)** Microscopic appearance of each strain. Scale bars represent 100 μm. **(B,C)** The average number of food vacuoles formed in each *Paramecium* strain. *Paramecium* were incubated with microbeads for 30 min and the number of food vacuoles in 20 *Paramecium* cells were counted. Data are the averages based on triplicate samples from three identical experiments and error bars represent standard deviations. Significant differences compared with RB-1 are indicated by asterisks (*P < 0.05). Scale bars represent 100 μm. **(D)** The cell division rate of each *Paramecium* strain. With the number of cells at the start of culture defined as 100%, the cell numbers after 24 h are shown as relative values. Data are the averages based on triplicate samples from three identical experiments and error bars represent standard deviations.

First, we aimed to identify the strains of *Paramecium* that showed high digestion efficiency of *L. pneumophila*. Sixty strains of *Paramecium* were examined in the first screening infection assay (Figure 1), and 29 strains of *Paramecium* showed digestion of *L. pneumophila* Ofk308 to varying degrees. These 29 strains were selected for the second screening assay (Table 1). In the second screening infection assay, only two *Paramecium* strains, *P. multimicronucleatum* Y-2 and *P. multimicronucleatum* YM-25, caused remarkable reduction of the bacterial load (Table 1). Both Y-2 and YM-25 are larger in body size compared to other *Paramecium* strains, especially *P. caudatum* RB-1 (Figure 2A). RB-1 was employed as a control host strain in subsequent examinations, since RB-1 has been analyzed in our previous studies and its characteristics have been described in detail (Watanabe et al., 2016, 2018). Indeed, RB-1 showed no reduction of *L. pneumophila* in our screening infection assay (Table 1), corresponding to the results observed in our previous studies (Watanabe et al., 2016).

**TABLE 1:**
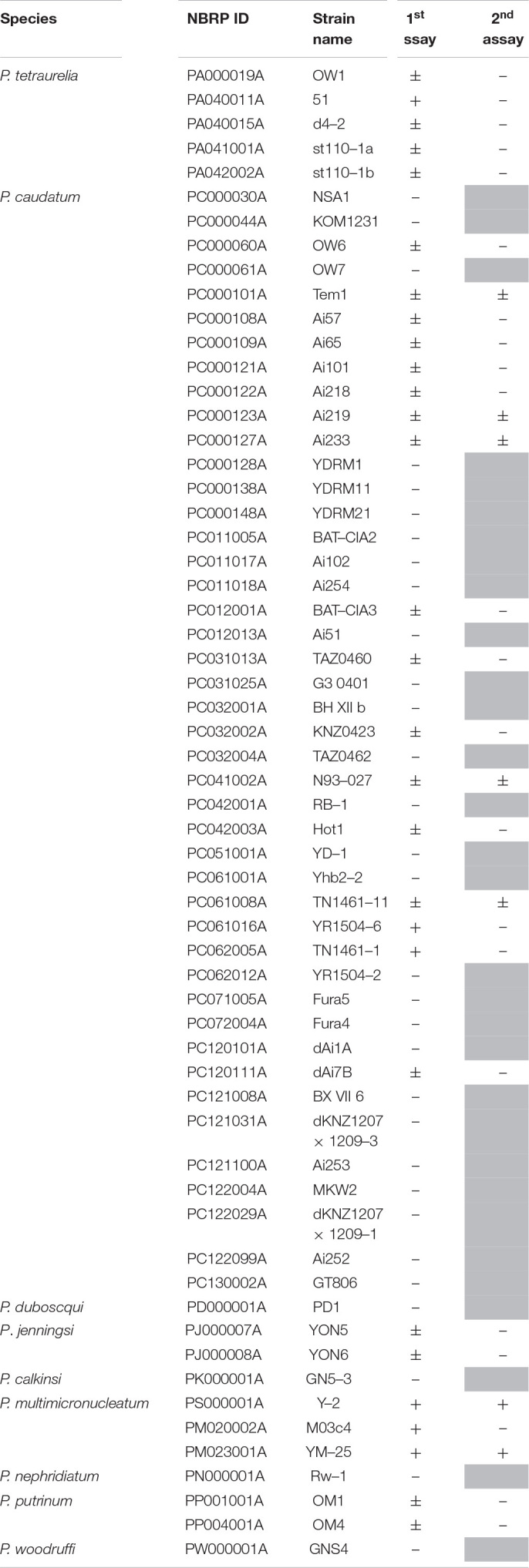
The results of screening assay to identify *Paramecium* strains that show digestion for *L. pneumophila* Ofk308.

*–; no reduction of bacterial load (50%–100%), ± ; low reduction of bacterial load (less than 50%), + ; high reduction of bacterial load (less than 10%) compared to control (cultured without *Paramecium*). The *Paramecium* strains which resulted in “ + ” or “ ± ” at 1^*st*^ screening assay were evaluated at 2^*nd*^ screening assay.*

The numbers of food vacuoles that formed intracellularly within 30 min in strains Y-2 and YM-25 were higher than the numbers formed in RB-1 (Y-2, 20.6 ± 1.56/cell, *P* = 0.0038; YM-25, 15.0 ± 0.62/cell, *P* = 0.028, versus RB-1, 12.7 ± 0.90/cell) (Figures 2B,C). A different degree of the numbers of food vacuoles was also observed in between Y-2 and YM-25. Although we expected the cell division rate to be linked to the digestion efficiency, when we determined the 24-hour division rates, there were no significant differences (*P* > 0.05) between the cell division rates of Y-2, YM-25, and RB-1 (Y-2, 232.56 ± 70.9%; YM-25, 247.56 ± 94.4%; RB-1, 170.67 ± 62.1%) (Figure 2D). The mobility of strains was also determined and no significant differences were observed (Supplementary Figure S2).

### Detailed Evaluation of Digestion Ability of Intracellular Bacteria

**FIGURE 3:**
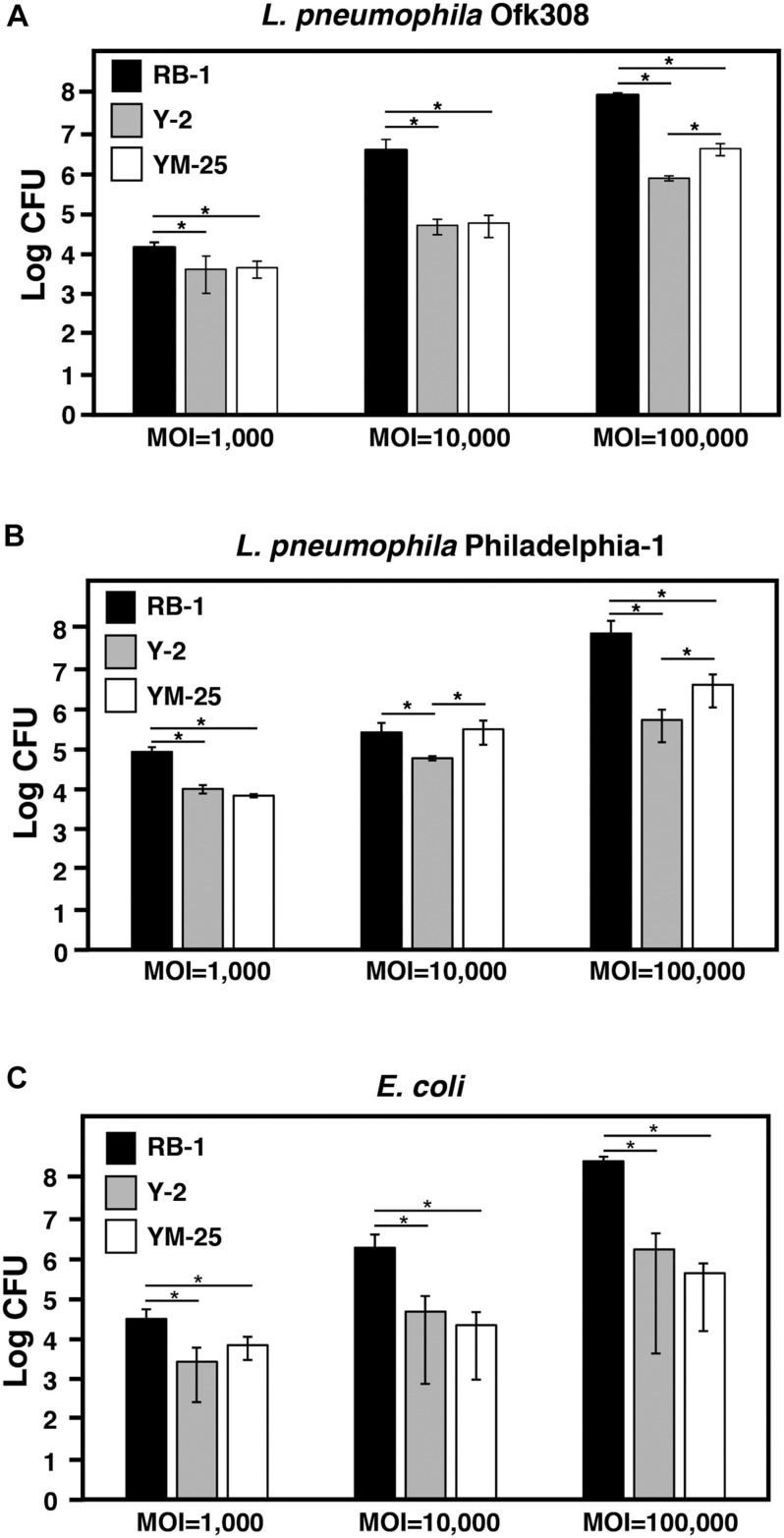
Evaluation of digestion of *L. pneumophila* or *E. coli* by RB-1, Y-2, and YM-25. RB-1, Y-2, and YM-25 were infected with *L. pneumophila* Ofk308 **(A)**, Phi-1 **(B)**, or *E. coli* **(C)** in 1.5 mL tubes at an MOI of 1,000, 10,000, or 100,000. Tubes were incubated at 25°C for 24 h. Samples were cultured on BCYE agar **(A,B)** or LB agar **(C)** after serial dilution to determine the CFU. Data are the averages based on triplicate samples from three identical experiments and error bars represent standard deviations. Significant differences are indicated by asterisks (*P < 0.05).

Next, we analyzed the number of intracellular bacteria in detail. Y-2, YM-25, and RB-1 were infected with *L. pneumophila* Ofk308 at various MOIs, and the CFU of *L. pneumophila* were counted 24 h after infection. At all MOIs, Y-2 and YM-25 showed larger bacterial reduction by digestion than RB-1. At an MOI of 100,000, Y-2 showed the largest digestion efficiency of the three strains (Figure 3A). We checked the digestion efficiency of other *L. pneumophila* strains to consider whether these results were confined to *L. pneumophila* Ofk308. Y-2, YM-25, and RB-1 were infected with *L. pneumophila* Phi-1 at various MOIs, and the Phi-1 CFU were counted 24 h after infection. Although the total number of Phi-1 CFU was different from that of Ofk308 in all *Paramecium* strains, there was no remarkable difference between the two strains of *L. pneumophila* (Figure 3B). We also performed the infection assay with *E. coli*, which is usually digested immediately by *Paramecium*. As a result, *E. coli* was also digested by Y-2 and YM-25 at a higher rate compared to the RB-1 strain (Figure 3C). These results indicate that Y-2 and YM-25 have a significantly higher digestive activity than RB-1, which is not specific to intracellular *L. pneumophila.*

### Analysis of *Legionella*-Containing Phagosomes

**FIGURE 4:**
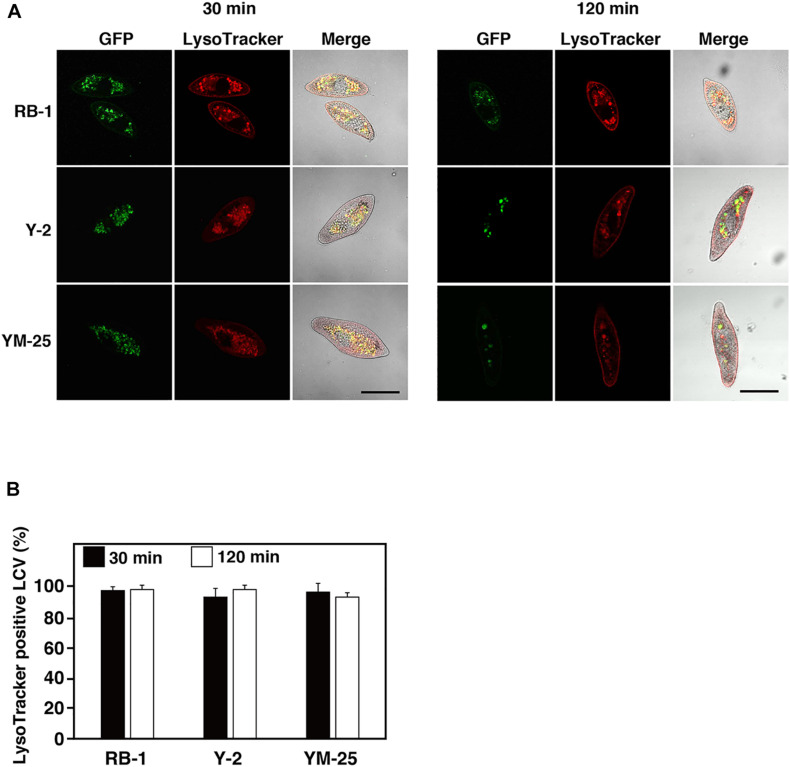
Maturation of *Legionella*-containing vacuoles (LCVs) in RB-1, Y-2, and YM-25 infected with *L. pneumophila* Phi-1. **(A)** LCV maturation 30 or 120 min after infection was evaluated using LysoTracker. *L. pneumophila* Phi-1 was added to RB-1, Y-2, and YM-25 at an MOI of 10,000. Scale bars represent 100 μm. **(B)** Relative LysoTracker-positive LCV percentages are shown, where the total of all LCVs is 100%. Data are the averages based on triplicate samples from three identical experiments and error bars represent standard deviations.

**FIGURE 5:**
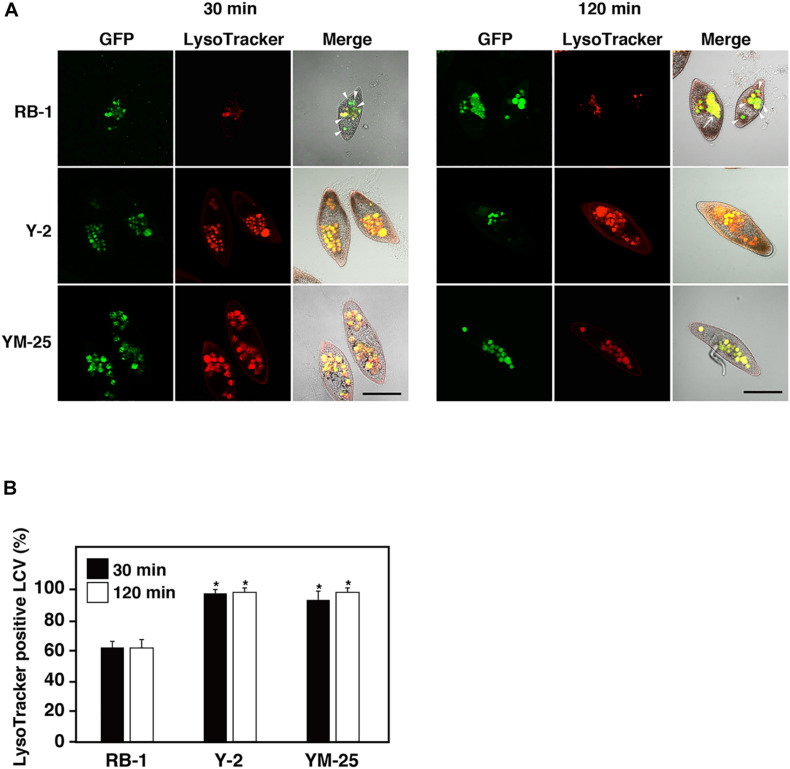
Maturation of LCVs in RB-1, Y-2, and YM-25 infected with *L. pneumophila* Ofk308. **(A)** LCV maturation 30 or 120 min after infection was evaluated using LysoTracker. *L. pneumophila* Ofk308 was added to RB-1, Y-2, and YM-25 at an MOI of 10,000. The arrowheads indicate LysoTracker-negative LCVs. Arrows indicate enlarged LCVs. Scale bars represent 100 μm. **(B)** Relative LysoTracker-positive LCVs percentages are shown, where the total of all LCVs is 100%. Data are the averages based on triplicate samples from three identical experiments and error bars represent standard deviations. Significant differences compared with RB-1 are indicated by asterisks (*P < 0.05).

To understand whether Y-2 and YM-25 show differences in their properties of food vacuole maturation during bacterial digestion, we evaluated the acidification and forms of *Legionella*-containing vacuoles (LCVs) using LysoTracker and GFP-expressing *L. pneumophila*. In our previous studies, it was confirmed that the acidification of LCVs in RB-1 is not inhibited during infection of *L. pneumophila* Phi-1 which established a stable intracellular relationship (Watanabe et al., 2016). The strain shows resistance against acidification of LCVs and survives within the LCVs. In this study, the LCVs of Y-2 and YM-25 also showed acidification during Phi-1 infection as with that of RB-1 (Figures 4A,B). Furthermore, we have previously reported that the cytotoxic-type *L. pneumophila* strain, Ofk308, partially inhibits LCV acidification and enlarges LCVs in RB-1. This modification of LCVs by Ofk308 is likely to correlate with cytotoxicity toward *Paramecium* hosts since the mutant strain of Ofk308 which shows no cytotoxicity toward *Paramecium* hosts failed to modify the LCVs. In the Y-2 and YM-25 strains, Ofk308 did not produce modifications of LCVs, such as inhibition of acidification and enlargement, during infection (Figures 5A,B).

## Discussion

The relationship between *L. pneumophila* and protist hosts could be an important factor in human infection. Many types of protist hosts, including *Paramecium*, which are thought to be reservoirs of *L. pneumophila* (Boamah et al., 2017), may exist ubiquitously in the environment (Foissner, 2007; Oliverio et al., 2020). In addition to *L. pneumophila*, relationships between protist hosts and intracellular bacteria, such as *Holospora* in *Paramecium* and *Mycobacterium* or *Neochlamydia* in *Acanthamoeba*, have been reported (Amann et al., 1991; Fokin et al., 1996; Cirillo et al., 1997; Horn et al., 2000; Goy et al., 2007; Fujishima and Kodama, 2012). The ecology and mechanisms of these intracellular relationships remain largely unclear. Exhaustive research using numerous protist strains is necessary to reveal these perspectives. To this end, we employed many *Paramecium* strains obtained from the NBRP to analyze the intracellular relationships between *Paramecium* and *L. pneumophila*.

In this study, we performed screening infection tests using 60 strains of 10 species of *Paramecium* host. As a result, we identified two strains of *Paramecium* which fail to establish the intracellular relationship with *L. pneumophila* due to their potent digestion: *P. multimicronucleatum* Y-2 and *P. multimicronucleatum* YM-25. Since another *P. multimicronucleatum* strain, M03c4, did not show this digestion of *L. pneumophila*, it appears that there is no tendency of digestion within this species and that the ability of digestion may not depend on species. Their responses to intracellular *L. pneumophila* are likely to be largely attributable to each strain’s characteristics. These results were also observed in our previous study (Watanabe et al., 2016). The syngen and mating type of Y-2 has not yet been classified, thus the relationship or affinity between Y-2 and YM-25 is unknown. Y-2 and YM-25 display a seemingly larger body size compared to other *Paramecium* strains (Figure 2A) and, probably due to this feature, they are able to establish and maintain a large number of food vacuoles (Figures 2B,C). It is assumed that the first step of the digestion process is the uptake of extracellular components, including bacteria, and that active formation of food vacuoles becomes an advantage for the effective digestion of bacteria. However, considering that there was a difference in the level of formation of food vacuoles between in Y-2 and in YM-25, this aspect may contribute to the effective digestion partially. Be that as it may, since we have been able to obtain and examine only three strains of *P. multimicronucleatum* in this study, it is impossible to conclude this issue here. In the further study, a comparative analysis of each strain’s characteristics using more *P. multimicronucleatum* strains would reveal it.

During the initial stage of screening, we expected that the high digestion efficiency of Y-2 and YM-25 was specific to *L. pneumophila*; however, they showed same level of digestion for *L. pneumophila* Ofk308 and Phi-1 strains in a relatively short amount of time (Figures 3A,B). These results indicate that Y-2 and YM-25 are able to reduce intracellular bacteria due to inhibition of some mechanisms which are involved in establishment of the intracellular relationship or due to complicated systems which control the intracellular growth of *L. pneumophila.* In mammalian hosts, *Legionella*-replication permissive cells are also restricted. While human cell lines such as THP-1 and HeLa have been used for *Legionella* research (Daisy et al., 1981; Takemura et al., 2000), in mice, their capability as a host of *Legionella* depends on their strain (Yamamoto et al., 1992; Losick et al., 2009). Macrophages isolated from *naip5* mutant A/J mice are recognized as a convenient *Legionella* host model (Yoshida et al., 1991; Wright et al., 2003). In these infection models, both the host and bacteria factors that are involved in intracellular growth have been identified and studied in detail (Yoshida et al., 1991; Lightfield et al., 2008; Vinzing et al., 2008). If such critical factors of *Paramecium* are identified in further analysis, they could explain the mechanisms involved in the high digestion efficiency for *L. pneumophila* in Y-2 and YM-25. Comparative genomic analysis of Y-2, YM-25, and RB-1 would also be helpful to reveal the differences in intracellular relationship with *L. pneumophila*.

Similar levels of digestion efficiency were also shown to *E. coli* in Y-2 and YM-25 (Figure 3C). These results suggest that both Y-2 and YM-25 have a high, non-specific digestion ability for a wide variety of bacteria. Although the strain of *E. coli* used in this study is not an intracellular bacterium and is digested promptly by *Paramecium*, the total bacterial number of *E. coli* was similar to that of *L. pneumophila*, especially for the Phi-1 strain. These results may be due to *L. pneumophila*’s failure to grow in culture medium for *Paramecium* at 25°C (Watanabe et al., 2018), while *E. coli* grows well in these conditions. The extracellular growth of *E. coli* may therefore mask the difference between *L. pneumophila* and *E. coli*.

We have previous confirmed that the acidification of LCVs occurs normally during *L. pneumophila* Phi-1 infection in *Paramecium* (Watanabe et al., 2016). The maturation of food vacuoles can be observed as LysoTracker-positive LCVs. It seems that *L. pneumophila* showing the stable intracellular relationship in *Paramecium* can withstand acidification and resist digestion, and then establish intracellular niches inside *Paramecium* (Watanabe et al., 2016, 2018). The process of establishing intracellular relationships in *Paramecium* is different from that in mammalian macrophages (Vogel and Isberg, 1999; Hubber and Roy, 2010). In addition, *L. pneumophila* inhibits acidification of LCVs and induces enlarged LCVs characteristically, and finally, results in the appearance of cytotoxic effects for *Paramecium* hosts (Watanabe et al., 2016). Analysis of the process of LCV maturation would help to reveal the relationships between *L. pneumophila* and *Paramecium*. In the present study, YM-25 and Y-2 did not show inhibition of LCV acidification or enlargement of LCVs (Figure 5A). Their LCV maturation processes were similar to those of stable intracellular relationship-type combinations such as *P. caudatum* RB-1 infected with *L. pneumophila* Phi-1. Our LysoTracker assay revealed no discriminative LCVs. YM-25 and Y-2 may have other mechanisms that can explain their high digestion efficiency for bacteria.

## Conclusion

We have revealed the peculiar *Paramecium* strains which do not establish intracellular relationships with *L. pneumophila*. Our results imply that not all *Paramaecium* hosts are able to accommodate and establish a stable intracellular relationship with *L. pneumophila*. The mechanisms and factors which dictate the success of the stable intracellular relationship between *Paramecium* and *Legionella* should be studied in detail in further studies. However, taking in another perspective, these *Paramecium* hosts can eliminate harmful or pathogenic bacteria, including *Legionella*, from environmental water. It may be possible that these *Paramecium* can be utilized in industrial water clarification applications and play a part in a novel system to control waterborne diseases. We are looking into the applications that may be developed from the results shown in this study.

## Data Availability Statement

The original contributions presented in the study are included in the article/Supplementary Material. Further inquiries can be directed to the corresponding author.

## Author Contributions

KW and MW conceived and designed the experiments. KW, YH, MS, and MW performed the experiments. KW, TS, and MW analyzed the data. MT and MF contributed reagents, materials, and analysis tools. KW and MW wrote the manuscript. All authors contributed to the article and approved the submitted version.

## Conflict of Interest

The authors declare that the research was conducted in the absence of any commercial or financial relationships that could be construed as a potential conflict of interest.

## Footnotes

1 http://nbrpcms.nig.ac.jp/paramecium/?lang=en

## Supplementary Material

Supplementary Figure 1 The assumed relationships between host *Paramecium* and *L. pneumophila*. We have previously identified a stable intracellular relationship; *Legionella* is able to avoid the digestion and establish the intracellular relationship inside *Paramecium* food vacuoles. We have also identified the case that the intracellular relationship is unstable; intracellular *Legionella* kills host *Paramecium* and fails to establish the intracellular relationship. We hypothesized that there is another case that the intracellular relationship is not established because of digestion of *Legionella* by *Paramecium* hosts.

Supplementary Figure 2 The swimming velocities of RB-1, Y-2, and YM-25. **(A)** Photomicrographs of swimming loci of RB-1, Y-2, and YM-25. Scale bar represents 500 μm. **(B)** The swimming velocities of RB-1, Y-2, and YM-25. The loci of 20 cells were measured in each experiment. Data are averages based on three identical experiments and error bars represent standard deviations.
