# Supplementary material for: Peculiar Paramecium Hosts Fail to Establish a Stable Intracellular Relationship With Legionella pneumophila
Source: Front Microbiol. 2020 Oct 23;11:596731. doi: 10.3389/fmicb.2020.596731 (PMC7644925; doi:10.3389/fmicb.2020.596731)
Supplement: Supplementary Figure 1 — The assumed relationships between host Paramecium and L. pneumophila. We have previously identified a stable intracellular relationship; Legionella is able to avoid the digestion and establish the intracellular relationship inside Paramecium food vacuoles. We have also identified the case that the intracellular relationship is unstable; intracellular Legionella kills host Paramecium and fails to establish the intracellular relationship. We hypothesized that there is another case that the intracellular relationship is not established because of digestion of Legionella by Paramecium hosts. [file Image_1.pdf]

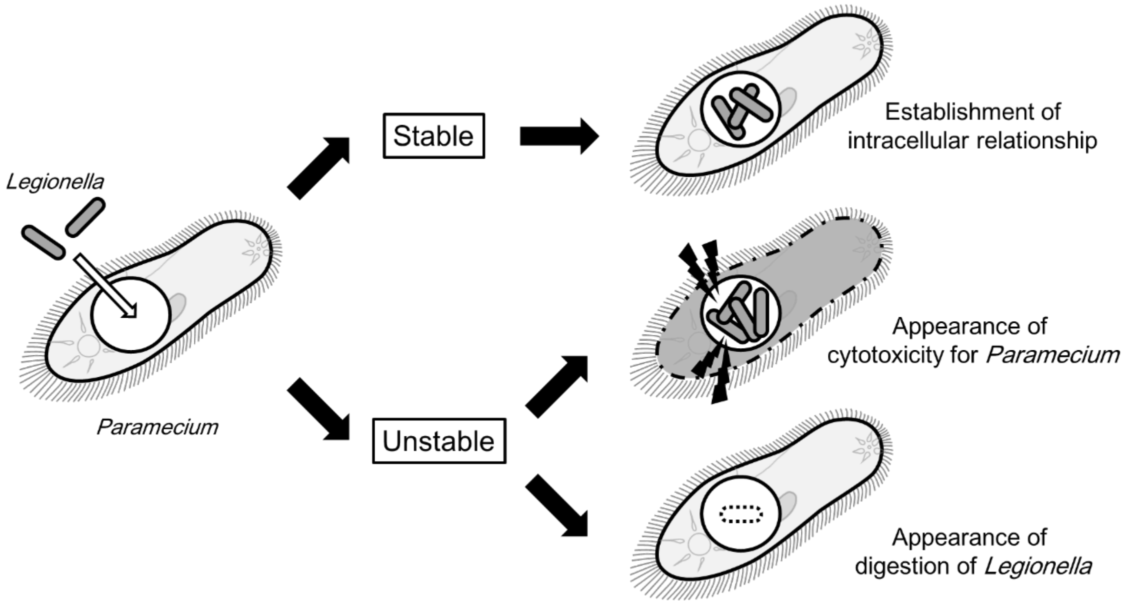

Supplementary Figure S1. The assumed relationships between host *Paramecium* and *Legionella*.

We have previously identified a stable intracellular relationship; *Legionella* is able to avoid the digestion and establish the intracellular relationship inside *Paramecium* food vacuoles. We have also identified the case that the intracellular relationship is unstable; intracellular *Legionella* kills host *Paramecium* and fails to establish the intracellular relationship. We hypothesized that there is another case that the intracellular relationship is not established because of digestion of *Legionella* by *Paramecium* hosts.

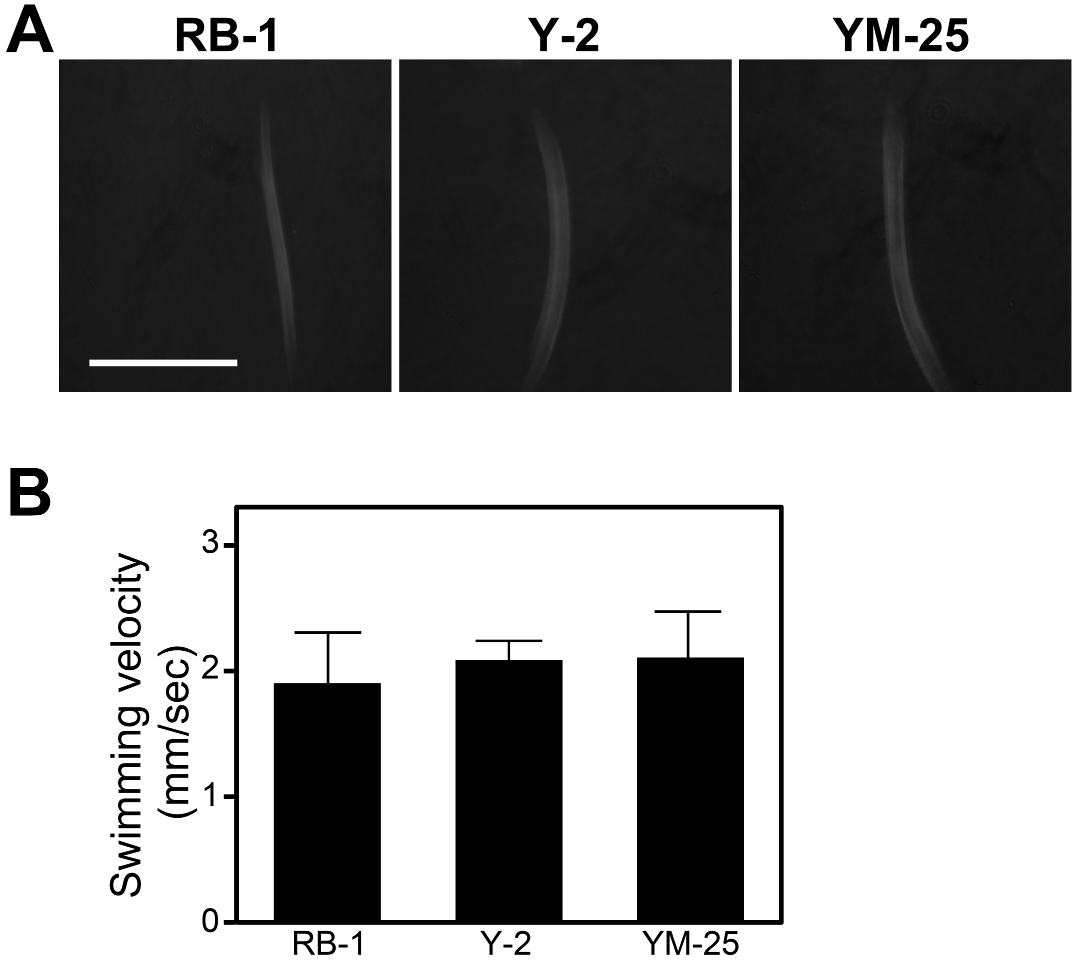

Supplementary Figure S2. The swimming velocities of RB-1, Y-2, and YM-25.
(A) Photomicrographs of swimming loci of RB-1, Y-2, and YM-25. Scale bars represent 500 μm. (B) The swimming velocities of RB-1, Y-2, and YM-25. The loci of 20 cells were measured in each experiment. Data are averages based on three identical experiments and error bars represent standard deviations.
